# From salt to hypertension, what is missed?

**DOI:** 10.1111/jch.14402

**Published:** 2021-11-30

**Authors:** Zhiyi Ma, Scott L. Hummel, Ningling Sun, Yuanyuan Chen

**Affiliations:** ^1^ Cardiology Department Beijing Tsinghua Changgung Hospital School of Clinical Medicine Tsinghua University Beijing China; ^2^ Ann Arbor Veterans Affairs Health System University of Michigan Frankel Cardiovascular Center Ann Arbor Michigan USA; ^3^ Cardiology Department Heart Center Peking University People's Hospital Beijing China

**Keywords:** absorption, excretion, hypertension, oral salt tolerance, sodium, storage

## Abstract

Excess salt intake is viewed as a major contributor to hypertension and cardiovascular disease, and dietary salt restriction is broadly recommended by public health guidelines. However, individuals can have widely varying physiological responses to salt intake, and a tailored approach to evaluation and intervention may be needed. The traditional sodium related concepts are challenging to assess clinically for two reasons: (1) spot and 24‐hour urine sodium are frequently used to evaluate salt intake, but are more suitable for population study, and (2) some adverse effects of salt may be blood pressure‐independent. In recent years, previously unknown mechanisms of sodium absorption and storage have been discovered. This review will outline the limitations of current methods to assess sodium balance and discuss new potential evaluation methods and treatment targets.

## 1 INTRODUCTION

Dietary salt restriction is recommended for many individuals, especially in older persons, those with hypertension, diabetes, metabolic syndrome, and chronic kidney disease, and others who are particularly susceptible to the effects of sodium on blood pressure (BP).^1^, ^2^, ^3^, ^4^ The World Health Organization (WHO) recommends salt intake of less than 5 g per day for adults and even less for children.^5^ The European Society of Cardiology (ESC) and the European Society of Hypertension (ESH) 2018 guideline recommended sodium intake limited to approximately 2.0 g per day (equivalent to approximately 5.0 g salt per day) in the general population and to try to achieve this goal in all hypertensive adults.^6^ The American College of Cardiology (ACC) and American Heart Association (AHA) 2017 guideline recommended that the optimal sodium intake was less than 1500 mg/day, and to aim for at least a 1000 mg/day reduction in most adults.^7^ According to the 2018 Chinese guidelines for the management of hypertension, sodium intake should be less than 2400 mg/day (approximately 6.0 g salt per day).^8^

Two randomized lifestyle intervention trials, the Trials Of Hypertension Prevention phase I (TOHP I) and phase II (TOHP II), found that dietary salt reduction over 18–48 months reduced BP.^9^, ^10^ After ten to fifteen years of follow‐up, patients who were originally assigned to lower salt intake had a 25% lower risk of major cardiovascular events.^11^ Based on the coronary heart disease (CHD) policy model of U.S., Bibbins‐Domingo and associates found that modest reductions in dietary salt (1.0‐3.0 g salt reduction) could substantially reduce cardiovascular events and medical costs.^12^ Together with several other clinical trials,^13^ these evidences support salt reduction to become a public health target. However, several prospective cohort studies revealed a J‐curve association of salt intake and cardiovascular disease or mortality,^14^, ^15^, ^16^, ^17^, ^18^, ^19^, ^20^, ^21^, ^22^ that is, higher event rate at both high and low intake. The Prospective Urban Rural Epidemiology (PURE) study used morning spot urine to calculate 24‐hour sodium excretion; its results supported a higher intake range, sodium 3–6 g/day (salt 7.6‐15.2 g/day), than currently recommended.^23^

As evidenced by these discrepancies, the optimal threshold for population sodium intake is not clearly established. This uncertainty stems from two key issues: (1) the physiological responses to changes in sodium intake can vary widely, and (2) sodium intake is challenging to assess due both to measurement limitations and patient‐based factors. An individualized recommendation for sodium intake would be ideal, but would require a clinically feasible, outcome‐relevant measure that incorporates sodium intake, storage, and excretion.

## 2 SALT SENSITIVITY AND HYPERTENSION

Controlled studies have identified subjects with a salt‐sensitive BP phenotype, that is, those in whom BP is higher during high‐sodium intake than during low‐sodium intake, and conversely those with salt‐resistant BP, whose responses are minimal or even depressor.^24^, ^25^, ^26^ The frequency of BP salt sensitivity is 32–64% in hypertensives and up to 50% in normotensives.^27^ The salt sensitivity of BP is derived by the interaction among heredity, comorbidities and environment.^27^ Genetic variants related to the salt sensitivity of BP involve several biological pathways linked to hypertension, such as the renin angiotensin aldosterone system (RAAS), sympathetic nervous system, ion and water channels, transporters and exchangers, endothelial system, intracellular messengers and the natriuretic peptide system.^28^ Age, gender, race/ethnicity, lifestyle (potassium intake, diet style, and physical activity) and comorbidities, such as obesity and chronic kidney disease, all contribute to BP salt sensitivity.^28^, ^29^, ^30^ Several studies link BP salt sensitivity and end organ damage, cardiovascular events, and long‐term mortality not only in hypertensives, but also in normotensives.^31^, ^32^, ^33^, ^34^

The American Heart Association, in a recent position paper, has affirmed BP salt sensitivity as an independent cardiovascular risk factor.^35^ Describing the phenotype requires BP measurement during manipulation of sodium balance and a threshold BP change that defines salt‐sensitivity. Salt‐resistant subjects may have a sub‐threshold increase of BP after salt loading, both hypertensive and normotensive persons can be salt sensitive or salt resistant, and several different blood pressure thresholds have been proposed. Neither of the two most common measurement methods, the acute saline loading test and chronic salt loading test, have acceptable feasibility for population study or reproducibility for routine clinical measurement in individuals.

Thus, salt sensitivity is mostly used in mechanism studies and epidemiological cohort end‐point analysis studies, limiting this concept's influence on risk assessment or development of treatment approaches.

## 3 ESTIMATING DIETARY SALT INTAKE

Accurate estimation of dietary intake is essential to determine the true impact of sodium reduction strategies. Food Frequency Questionnaires, Likert‐scale estimations of how frequently common foods are consumed, or food diaries, which involve direct recording of food intake, are the most common methods used.^36^, ^37^, ^38^ Of these techniques (listed in Table 1), 24‐hour dietary recall or variants such as a 2‐ or 3‐day recall are likely more accurate than questionnaires for daily intake. Mobile application‐based diary programs and artificial intelligence techniques, such as image recognition, has improved the convenience of these evaluations. However, the limitation of nutrient content databases, over‐ or underestimations of food amount, and other systematic and random errors can greatly influence the accuracy of these methods and limit their clinical application.^39^

**TABLE 1:** Methods for dietary salt intake estimation

Methods	Examples	Features
Food frequency questionnaires	Likert‐scale estimations	Time sonsumint
		Not accurate
Food diaries	24‐hour dietary Recall	Convenient
	2‐ or 3‐day recall	Limitation of nutrient content databases
	Artificial intelligence‐based diary	
24‐ hour urine sodium	–	Gold standard
		Inconvenient
Spot urine sodium	INTERSALT equation	
	Kawasaki formula	Underestimates at high level salt intake
	Tanaka formula	Overstimates at low level salt intake

Nearly 95% of ingested sodium is excreted through urine, and historically 24‐hour urine sodium has been assumed to be equal to sodium intake. Many cohort studies of the relationship between sodium and cardiovascular diseases use urine sodium methods to estimate intake. In 1980s, the INTERSALT study established the gold standard position of 24‐hour urine sodium.^40^, ^41^, ^42^ Spot morning urine sodium has been considered to replace the gold standard because of its convenience.^43^

The INTERSALT equation, Kawasaki formula and Tanaka formula are the most commonly used calculation formulas to back‐calculate 24‐hour urine sodium from spot samples.^44^ Unfortunately, when compared to 24‐hour urinary sodium, spot urine sodium often underestimates intake at high levels and overestimates intake at low levels no matter when the spot sample is collected.^44^, ^45^, ^46^ These inaccuracies in the specific range of clinical concern severely compromise the value of spot urine for estimating sodium intake. Therefore, spot urine is best suited for population study, not individual evaluation.^44^

In addition to imposing a significant burden on patients, 24‐hour urinary collection is not as accurate a measurement of sodium intake as previously believed. According to the criteria established by Cobb et al, only 24‐hour urine collections following a specific study protocol with quality assurance or incomplete collection exclusion have lower risk of systematic error.^39^ However, in a long‐term study of healthy young men on controlled diets with measured consumption, Lerchl and associates found that single 24‐hour urine collections at salt intakes ranging from 6 to 12 g/day were not suitable to detect a 3‐g difference in individual salt intake.^47^ Although repeated measurements of 24‐hour UNaV improve precision, this is not a feasible approach for routine clinical measurement.

## 4 SODIUM ABSORPTION, STORAGE, AND “BLACK BOX”

**FIGURE 1:**
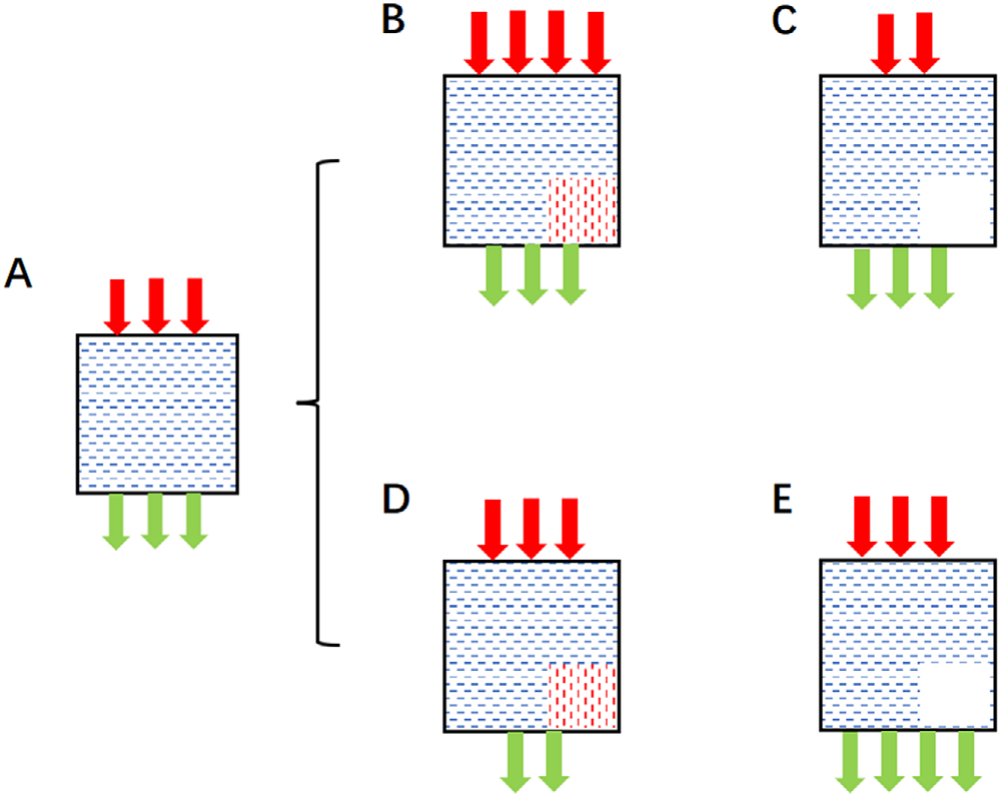
Sodium intake, sodium excretion, and “black box.” Red arrow: sodium intake; green arrow: sodium excretion; black square with blue dots: black box, meaning sodium in body. Red dots: meaning more sodium left in the body. Empty small square: meaning less sodium left in the body

If even meticulously collected urine samples with extensive quality control are this inaccurate, this strongly challenges the principle that 24‐hour urinary sodium excretion is equivalent to intake. Since nearly 95% of ingested sodium is excreted through urine, non‐renal sodium losses are unlikely to be the major source of error under most circumstances. This raises two possibilities: (1) sodium absorption from the gastrointestinal (GI) tract is variable, and/or (2) not all absorbed dietary sodium is excreted within 24 hours. These unknowns are illustrated in Figure 1, with red arrows representing sodium intake, green arrows sodium excretion, and the “black box” depicting the amount of sodium in the body.

In Figure 1, panel A, B, and C represent the same sodium output (urine sodium excretion) with different sodium input (sodium intake), while there is more sodium left in “black box” (the body) for panel B and less for panel C. Panel D and E have the same input as panel A but different output, and there is more sodium left in the “black box” for panel D and less for panel E.

With equivalent output in panels A, B, and C, it is hard to distinguish sodium input difference and more or fewer sodium left in “black box.” Compared with panel A, the decreased sodium output in panel D seems to represent sodium intake reduction, but actually reflects more sodium left in the body based on equivalent sodium input. The increased sodium output in panel E would normally be interpreted as increased sodium consumption, but instead reflects increased sodium excretion based on the same sodium input as panel A. The “gold standard” 24‐hour urine sodium can only represent itself since it has to pass through “black box” to represent sodium intake.

**FIGURE 2:**
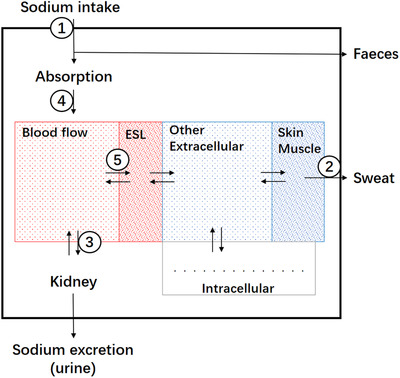
Sodium absorption and storage in “black box.” ESL, endothelial surface layer. Step 1–5 represent different steps involving sodium absorption, storage and excretion. Step 1,2 and 3 represent traditional sodium intervention pathways, including oral sodium reduction, physical exercise, and diuretics, respectively, and natriuresis of SGLT2 inhibitor (sodium‐dependent glucose transporters 2 inhibitor) is at step 3. Step 4 and 5 represent new sodium intervention pathways, such as colonic bacteria balancing, tenapanor, and sulodexide. Partially adapted from.^59^

The absorption, processing, and excretion of sodium are illustrated in Figure 2. Two processes are particularly critical to understand the “black box” in Figure 1. The first process is sodium absorption after salt intake (Step 4 in Figure 2). The proximal colon is the main site of dietary ion absorption.^48^, ^49^, ^50^ The regulation of sodium absorption in mammalian proximal colon occurs at the apical surface of epithelial cells. There are two cellular mechanisms, Na+/H+ and Cl−/HCO3− exchangers (NHE3)^51^ and epithelial sodium channel (ENaC).^52^ Sodium absorption in colon is an energy consuming process mediated by ATPase.^53^, ^54^ Butyrate and other short‐chain fatty acids (SCFA), energy sources produced by colonic bacteria, influence numerous colonic epithelial cell functions and play an important role in regulating colonic sodium absorption.^55^, ^56^ An early study from Tumamian and associates revealed mineralocorticoid receptor and glucocorticoid receptor mediated changes in sodium transport in rat colon.^57^ Richards and associates provided more evidence on gut local renin angiotensin aldosterone system and its regulating role of sodium uptake from colon.^58^

In recent years, the relationship between intestinal bacteria and hypertension has of increasing interest. Sodium can affect the gut microbiome and thus induce pro‐inflammatory and immune responses, such as interleukin‐17 (IL‐17) production. One of the mechanisms of IL‐17 is to induce endothelial dysfunction and to increase renal sodium reabsorption, which may contribute to salt sensitive hypertension.^58^ There is little evidence on whether reverse action on sodium absorption in colon exists from these immune responses. It is also unclear whether colonic bacteria could affect sodium absorption in colon through production of SCFA. Further studies are needed to understand the complicated crosstalk between the gut microbiome and sodium absorption/excretion within individuals.

Historically, a two‐compartment model of body sodium was used to explain sodium storage and sodium homeostasis. Recently, sodium storage in a “third compartment” has been confirmed in several studies (Figure 2).^59^ One site of nonosmotic sodium storage is skin interstitium and skeletal muscle. A large amount of sodium with concentration up to 180–190 mmol/L can be found in the skin interstitium without influence on extracellular volume.^60^ By coupling ^23^Na‐MRI with traditional ^1^H‐MRI, it is possible to evaluate sodium accumulation in the skin and muscles.^61^ Higher concentrations of sodium have been documented in skeletal muscles and skin of animals with experimental hypertension and in hypertensive patients than that in the plasma.^62^ Sodium accumulation in skin interstitium is induced by increased content and sulfation of negatively charged glycosaminoglycans (GAGs), which bind and inactivate sodium. Interstitial sodium storage has several influencing factors, such as age, gender, salt intake, blood pressure, diabetes, infection, and inflammation.^61^, ^63^, ^64^, ^65^, ^66^, ^67^, ^68^, ^69^, ^70^ The other major nonosmotic sodium storage is the endothelial surface layer (ESL). The ESL is a dynamic layer on the luminal side of vascular endothelial cells which continuously exchanges with flowing blood and similarly buffers circulating sodium with negatively charged GAGs.^71^ When ESL is perturbed, more sodium ion is transported into and between endothelial cells, leading to endothelial stiffening and water retention.^60^, ^72^ In addition, other endothelial cell functions are influenced by perturbed ESL, such as nitric oxide (NO) production.^73^ Based on the phenomenon that the surface layer of erythrocytes “mirrors” the ESL, a salt blood test (SBT) and its simplified test (SBT‐mini) were developed representing red blood cell sodium buffering capacity and indirectly representing ESL condition and even salt sensitivity.^74^

Treatments such as diuretics, sodium glucose cotransporter 2 (SGLT‐2) inhibitor and dialysis can all influence interstitial sodium content.^63^, ^75^, ^76^ Based on recent studies, skin sodium accumulation is hypothesized to be harmful and found to involve with vascular function,^77^ left ventricular hypertrophy,^78^ heart failure, hyperaldosteronism, and acute kidney injury.^61^, ^63^, ^75^, ^79^ However, whether skin sodium accumulation is the cause or is the parallel organ damage still need further investigation. Conversely, ESL is considered to have a barrier or buffering function for excess sodium, especially immediately after salt intake. Based on large vascular system, ESL for sodium storage has a volume of 1.5 L in healthy persons and significant amounts of sodium can be accumulated right after sodium has been absorbed into the circulatory system.^80^

These underappreciated mechanisms of sodium absorption and storage could help explain the “black box” in Figure 1 and Figure 2. With further investigation of these mechanisms, both sodium absorption and storage may provide novel biomarkers, targets, or strategies for sodium‐related disease prevention and management.

## 5 INDIVIDUAL, EACH DAY, AND EACH MEAL

**FIGURE 3:**
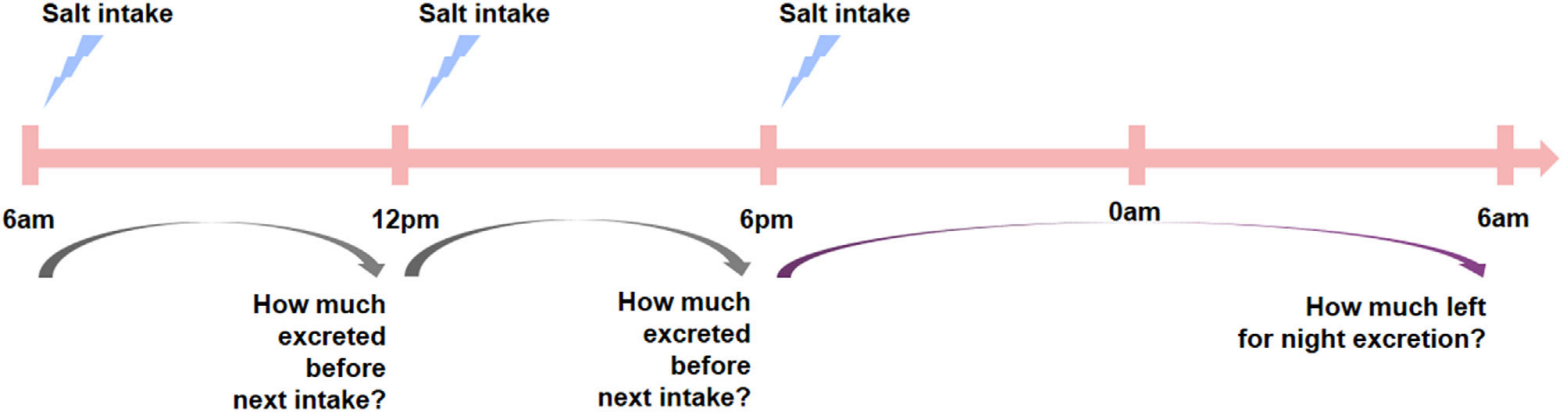
Simulation diagram salt intake from each meal during one day

The progression from salt to cardiovascular disease results from chronic pathophysiological effects. The core principle of salt reduction is to balance intake and excretion to avoid target organ damage. Several questions arise when focusing on salt intake from one meal. How long is needed to excrete the salt load? How much be excreted in a whole day? How much sodium can be excreted before the next intake, and what happens if excretion is not complete beforehand? How much sodium remains after food intake for excretion overnight? (Figure 3.) Are there different individual abilities for these? What influences this individual ability from the “black box,” and how do these individual differences affect the continuum from salt to hypertension and cardiovascular disease?

The response to saline infusion may provide clues, although it is not a physiological salt intake pathway. Drummer and associates found that the largest increase in urinary sodium excretion was between 3  and 22 hours and that urinary sodium excretion rates significantly increased over two days after acute saline infusion.^81^ Lobo and associates observed that an additional oral glucose load did not affect urinary sodium excretion after rapid 2‐L 0.9% saline infusion.^82^ These studies suggest that measuring time course of urinary sodium excretion after oral saline intake might be feasible, especially after a standardized salt load. Nishimuta proved this principle by outlining the urinary sodium excretion time course over 4 hours after ingestion of 500 mL 0.9% saline and pure water.^83^

**FIGURE 4:**
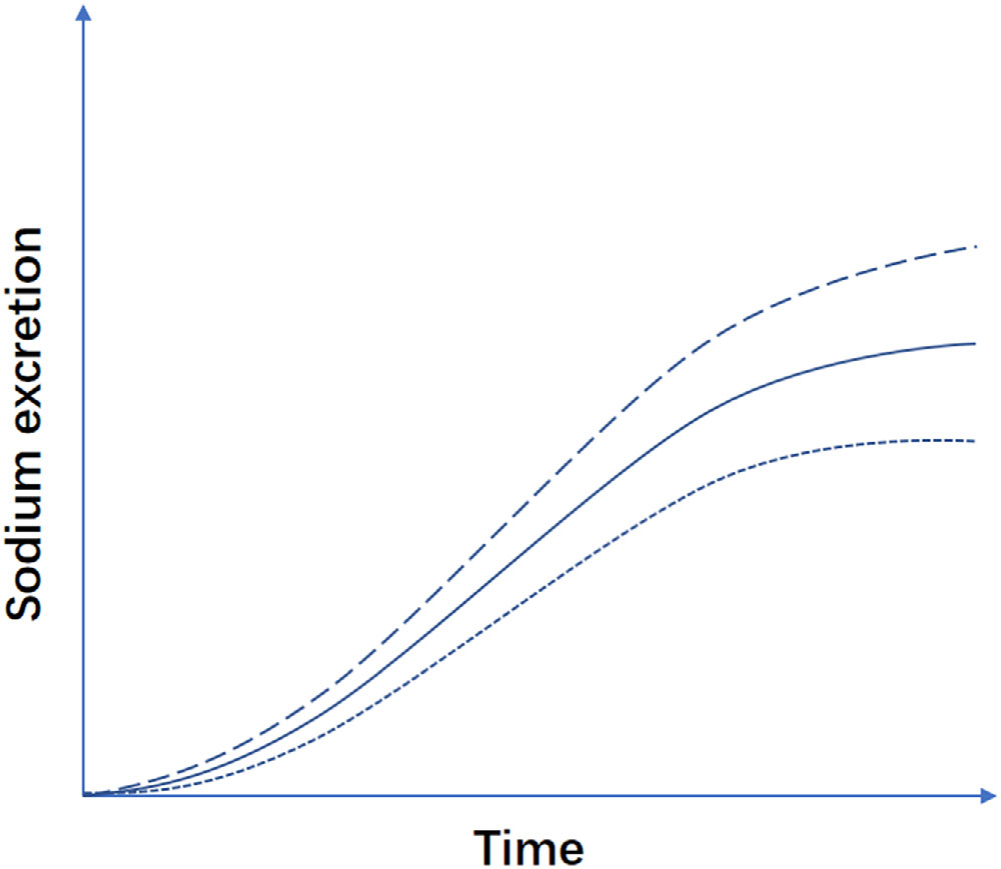
Hypothesis of sodium excretion after one standard salt intake. The lower dotted‐line represents downshift, eg, because of less sodium absorption, more sodium excreted in feces or sweat, or more sodium reabsorption. The upper dashed‐line represents upshifting because of better excretion or less reabsorption

In order to evaluate the ability to absorb and excrete salt after a standard salt ingestion, the hypothesis of oral salt tolerance is presented as Figure 4. We could hypothesize that sodium excretion curve might downshift because of less sodium absorption, more sodium excreted in feces or sweat, or more reabsorption while upshifting because of better excretion or less reabsorption.

We have established a new method, “Oral Salt Tolerance Test (OSTT)” to evaluate these mechanisms in combination.^84^ In short, the participant fasts after 9 pm and empties their bladder before sleep on the day before OSTT. In the morning of the test, the participant first empties their bladder and then drinks 500 mL 0.9% saline in 15 minutes. Urine volume and urine samples for sodium test are collected at 30 , 60 , 120, and 180 minutes time points. Our pilot data showed acceptable consistency of OSTT upon repeated measures for its first volunteer. The intraclass correlation coefficient for equal sodium chloride amount among three days tests was 0.94 (95% confidence interval 0.6810–0.9958). Another interesting result was that only less than 50% of salt was excreted through urine in three hours. Lobo and associates found that around 76 mmol sodium, approximately equal to the 4.5 g sodium chloride in 500 mL 0.9% saline, was excreted in six hours after rapid 2‐L 0.9% saline infusion.^82^ In Nishimuta's study, there was also less than 50% sodium excreted through urine in three hours after 500 mL 0.9% saline ingestion.^83^ Thus, these data were partially consistent with our pilot data. The details of the OSTT need further adjust, such as the optimal time points for sampling and other influencing parameters besides water volume or glucose. Nishimuta and Peniamina's studies provide information on potassium influence on sodium excretion.^83^, ^85^ Besides urine electrolytes, Bazzell and associates provided information on the relationship between urine sodium excretion and mRNA in urine supernatant which was mainly from extracellular vesicles, such as exosome and microparticles.^86^ While investigating the physiological and pathological meanings of OSTT in following studies, we should also recognize that OSTT is easy to operate and expected for application at different stages from healthy, sub‐health, prehypertension to hypertension, and from salt intake to hypertension during one's life.

**FIGURE 5:**
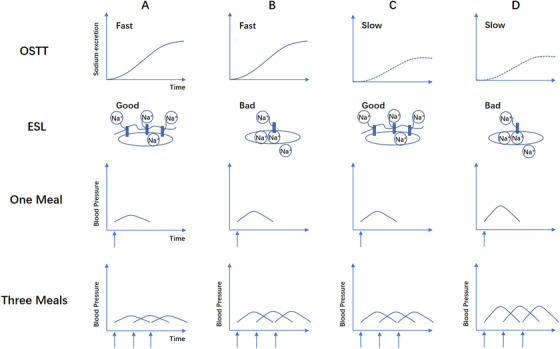
Hypothesis of different blood pressure variation based on different oral salt tolerance and ESL condition. OSTT, oral salt tolerance test; ESL, endothelial surface layer; Na^+^, sodium. Each arrow represents one meal. In one meal panel and three meals panel, each curve represents possible blood pressure change induced by sodium intake from each meal. Partially adapted from.^71^

Based on three meals in one day, from sodium intake to sodium excretion, OSTT might help us to understand BP variation and adjust hypertension chronotherapy in another way. As a package evaluating sodium excretion after a standardized salt intake, the OSTT result is the comprehensive effect of absorption, storage, reabsorption, and renal excretion and could help us to evaluate the whole function of the “black box.” For example, different OSTT results with different ESL condition might theoretically export different blood pressure phenotypes or patterns as shown in figure 5. Non‐dipping hypertension is generally believed to be a reflection of the need of nocturnal sodium excretion.^87^ When trying to restore normal dipping, we need to make clear what be the fundamental issues, to decrease sodium intake and storage and increase excretion during daytime or to change the timing of dosing to increase sodium excretion during nighttime.

Our traditional treatments on sodium are salt reduction and diuretics (Step 1 and 3 in Figure 2). If considering salt in sweat, physical exercise could be another method (Step 2 in Figure 2). Recently, tenapanor, a NHE3 inhibitor decreasing sodium absorption, is under evaluation for possible antihypertension function although it failed in a chronic renal disease and type 2 diabetes stage II clinical trial (Step 4 in Figure 2).^88^ Butyrate and other short‐chain fatty acids (SCFA) might also affect step 4 in figure 2 through ENaC pathway, although the role of the gut microbiome still needs confirmation. Sulodexide, a highly purified mixture of GAGs, could restore ESL.^89^ It has potential antialbuminuric effects^90^, ^91^ and blood pressure lowering effect,^92^ which require further investigation (Step 5 in Figure 2). SGLT2 inhibitors were originally developed as glucose‐lowering agents. However, one of their main effects is natriuresis (Step 3 in Figure 2), which might play an important role for its antihypertension effect and cardiovascular benefits.^93^

While looking for novel treatment targets, the focus must expand beyond BP effects of sodium to the balance of sodium in the “black box.” The physiological response to a single event of sodium ingestion may provide important individualized insights into the prevention or treatment of hypertension. This may extend beyond simply recommending dietary sodium restriction (or lessening the need for it). For example, based on this information diuretic treatment could be started, or the dose/timing adjusted more effectively. In combination with emerging markers of intrarenal physiology, microbiome composition, or ESL integrity, a holistic assessment of sodium balance could guide selection of other agents such as mineralocorticoid receptor antagonists, short‐chain fatty acids, or sulodexide (55‐58,89).

## 6 CONCLUSION

Dietary sodium restriction is generally believed to prevent hypertension and associated cardiovascular diseases. However, guideline recommendations vary, dietary sodium intake is challenging to measure, and the traditional renal‐dominant paradigm of sodium processing has recently been questioned. A more holistic evaluation, incorporating sodium absorption, storage, and excretion, is needed to understand sodium related day‐by‐day or chronic pathophysiological changes. This approach could individualize dietary advice and identify patient‐specific targets for novel strategies to prevent and manage hypertension and its complications.

## CONFLICTS OF INTEREST

No conflict of interest.

## AUTHOR CONTRIBUTIONS

Zhiyi MA contributed to the conception of the review and wrote the manuscript; Scott L. Hummel mainly contributed to the sodium absorption and storage parts and edited the whole manuscript; Ningling SUN contributed to the dietary salt intake estimation part of the manuscript; Yuanyuan CHEN contributed to the salt sensitivity and hypertension part of the manuscript.
